# Promoting the Learning Mindset Among Undergraduate Medical Students: A Qualitative Pilot Study on an Active Self-Learning Module Aimed at Openness During the Feedback Process

**DOI:** 10.1177/23821205261423394

**Published:** 2026-02-11

**Authors:** Véronique Lapierre, Anne-Charlotte Côté, Isabelle Burnier, Diane Bouchard-Lamothe

**Affiliations:** 1 Faculty of Medicine, 12365 University of Ottawa, Ottawa, Canada; 2 Francophone Affairs, Faculty of Medicine, University of Ottawa, Ottawa, Canada; 3 Institut du savoir Montfort, Ottawa, Canada

**Keywords:** Formative feedback, feedback reception, learning posture, inner speech, active self-learning module

## Abstract

**Background:**

After decades of feedback courses for supervisors, students now have access to courses on the interactional feedback process. However, this interactional feedback process requires students to be receptive to discussions in order to assimilate and apply the information shared. To achieve this, an active self-learning module (ASLM) offered students a reflective exercise and strategies based on the H.O.S.T. (humility, openness, shared explicitness and tenacity) behavioural model.

**Methods:**

The purpose of this pilot study was to explore how the ASLM shaped students’ understanding of the learning mindset within a sample of six students from the Francophone stream of the University of Ottawa MD Program. These students engaged in simulated clinicals and clinical rotations during which they receive feedback. Semi-structured interviews were used to explore students’ perceptions of the module and the self-reflection it sparked. The data were analyzed using a rigorous thematic analysis.

**Results:**

The thematic analysis identified two main themes and six sub-themes. Students perceived the module's reflective approach as promoting their engagement in student-supervisor interactions and the personal growth mindset necessary for emotional regulation. The importance of a two-way relationship with the supervisor was highlighted, raising the possibility of training intended for both supervisors and students.

**Conclusion:**

The ASLM introduced to a small sample of learners allowed them to reflect on their learning mindset and to discover strategies that could help them receive and engage in feedback more effectively. Although participants viewed this reflective exercise as a promising initial step in influencing attitudes and behaviors related to feedback, further exploration with a larger population is needed.

## Introduction

Formative feedback is essential to developing the clinical, professional and interpersonal skills needed to practise medicine safely for patients.^1–5^ Formative feedback is described as an exchange between supervisor and student with a view to improving the future physician's skills.^6^ This process requires the student to receive the feedback, ie, assimilate information from various sources, and apply the information received.^6^ Although feedback is viewed as a two-way process that is part of an educational alliance that actively involves students,^7,8^ courses on the topic are aimed primarily at supervisors.^3,9^ To compensate for this deficiency, the concept of developing students’ feedback literacy was operationalized.^10–14^ These courses aim to stimulate students’ ability to recognize, understand, produce and act on feedback received.^13^ Increasing feedback literacy among students has proven to be positive and has facilitated their engagement in the feedback process,^10–14^ but some intrapersonal deficiencies have been identified that were preventing students from engaging fully.^15^

Given that feedback is described as an emotionally-charged external stimulus,^16–19^ students may react differently in a learning setting, which is contrary to the formative goal of feedback.^20^ We know that medical students often exhibit a closed mindset related to the evaluator role, prompting them to do what it takes to *seem competent* rather than *become competent*, which limits the benefits of formative feedback sessions and skills improvement.^21,22^ The literature on feedback illustrates the importance of developing a personal growth mindset. This personal mindset, as described by Dweck (2008),^23^ is an asset that allows students to “see challenges and mistakes as learning opportunities” and to be more receptive to feedback,^7,23,24^ hence the suggestion of adopting the “learning posture.”^22,24,25^ This mindset facilitates emotion management, promotes open-mindedness and encourages self-evaluation and active participation in the feedback process.^22^ To successfully adopt the learning posture, Giroux and Girard focus on inner speech as an educational strategy.^22^ Agreeing that reflection^7,12,26^ and self-regulation processes are important in learning,^27^ Lee supports Vygotsky's position that presents inner speech as the basis of the mechanisms that regulate behaviours, thoughts and emotions crucial to learning and skill development.^27^ Although adopting the learning posture based on this educational strategy needs to be prioritized, few studies focus on how to achieve this.

To fill this gap, the University of Ottawa's Faculty of Medicine Office of Francophone Affairs has developed an active self-learning module (ASLM) offering students a reflective exercise based on the H.O.S.T. behavioural model.^28^ This model presents various strategies to make it easier to adopt the learning posture and to better receive feedback. This cognitive-behavioural model, which promotes inner speech, focuses on adopting attitudes (humility and open-mindedness) and behaviours (shared explicitness and tenacity) that support a personal growth mindset aligned with the learning posture and student engagement in the feedback process. Hoping to spark personal change in students, this model and this module focus on the precontemplation, contemplation and action stages cited in Prochaska and DiClemente's model of behaviour change.^29^

As such, we aimed to explore how the ASLM providing strategies to facilitate self-regulation was perceived by medical students in relation to their awareness of the learning mindset. We also sought to understand how it influenced their comfort in receiving feedback and participating in the feedback process with their supervisor.

## Methods

### Qualitative Approach and Research Paradigm

To achieve our research objectives, we used a latent thematic analysis within an interpretive research paradigm. This approach enables us to explore how the ASLM influenced the adoption of a leaning mindset and student's comfort in participating in the feedback process with their supervisor. The reporting of this study conforms to the Standards for Reporting Qualitative Research (SRQR) guidelines (Appendix 1).^30^

### Researcher Characteristics and Reflexivity

The research team consisted of four co-researchers with complementary background in medical education, qualitative research, and clinical training. The supervisor of this research project (DBL) is a learning advisor at the University of Ottawa's Faculty of Medicine Office of Francophone Affairs. She has extensive experience in ideation and development of academic resources, in qualitative research, and she is the co-author of the H.O.S.T. model. Co-investigators (VL and ACC) are third-year and fourth-year medical students. They facilitated the recruitment of participants and contributed objectively to all stages of the research project, although it is acknowledged that their experience as students may introduce confirmation bias. One co-researcher (IB) is a faculty member involved in undergraduate medical education with significant practical experience in training supervisors and learners. She has long overseen simulated clinicals for pre-clerkship medical students, with the goal of fostering clinical reasoning and skill development thought structured, constructive feedback provided by trained supervisors. No member of the research team had a supervisory or assessment role with respect to the students involved in the study.

### Context

This research was carried out within the Francophone stream of the University of Ottawa Faculty of Medicine MD program, and all data were collected outside regular class hours.

### Sampling Strategy

We chose the non-probability sampling method for practical reasons, as recommended in works by Murphy et al^31^ and Higginbottom.^32^ An invitation to participate in this research project was sent to medical students through a weekly email shared by the University of Ottawa medical students association (Aesculapian Society), between November 2023 and March 2024. All francophone students in the Faculty of Medicine's MD program were included in this study. No specific exclusion criteria based on gender, age, level of education or comfort with feedback were applied. Four students expressed interest in participating in the study, which was not enough to achieve potential data saturation, as described in works by Guest, Bruce and Johnson.^33^ Other recruitment efforts, mainly snowball sampling, helped recruit eight additional participants.^34^ Although 12 participants were initially recruited, only six chose to participate in the semi-structured interviews, which represent the final sample retained for the qualitative analysis. Therefore, data saturation could not be achieved within this sample.

### Ethical Issues Pertaining to Human Subjects

We conducted this study in accordance with the University of Ottawa's institutional ethics standards (# H-09-23-9527 – REG-9527). All participants in this research project signed a consent form prior to data collection, and they were assigned an identification number to respect anonymity and the confidentiality of their answers.

### Data Collection Methods and Instruments

**Figure 1.**
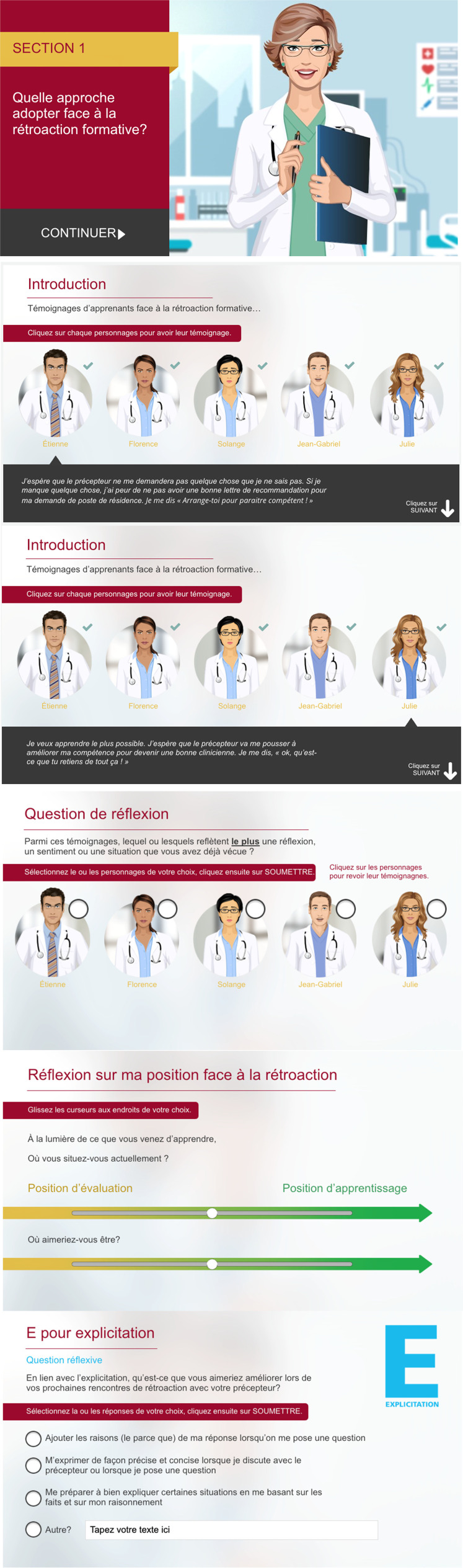
Overview of the active self-learning module “Being More Open to Formative Feedback *[Mieux accueillir la retroaction formative*],” and its five characters promoting introspection through identification. The training module is available in French, and can be accessed by contacting the corresponding author.

Individual semi-structured interviews with the six participants were conducted between January and March 2024, no later than 2 weeks after the individual completion of the ASLM. The ASLM entitled Mieux accueillir la rétroaction formative [Being More Open to Formative Feedback], based on the H.O.S.T. model, are both developed by the University of Ottawa's Faculty of Medicine, Office of Francophone Affairs. The aim of this 20-min-long virtual module is to (a) help students situate themselves in terms of their experience receiving feedback; (b) differentiate between the evaluation posture and the learning posture; (c) describe the key points of effective formative feedback; and (d) explain the four dimensions of the H.O.S.T. model.^28^ As they navigate the ASLM, students are asked to identify with one of five characters, all of whom approach feedback differently, ranging from a fixed mindset characterized by apprehension and closedness towards feedback to a mindset that embraces personal growth and continuous learning. (Figure 1) The ASLM is a digital resource based on introspection, designed to provide an active and engaging experience. Guided by a virtual tutor, learners are encouraged to self-explore and reflect on the content by answering questions, and selecting among various response options. (Table 1) If a required step is skipped, an alert message appears.

**Table 1.** List of Integration and Reflective Questions Proposed by the ASLM.

**Integration** Q**uestions** Let's go back to the testimonials from our five learners. Can you place them in relation to their position on formative feedback? What is the role of the mentor in formative feedback? What is the main element of the [H.O.S.T] model that Solange should work on to better receive feedback? **Reflective Questions** Which of these testimonials best reflect a thought, feeling, or situation you have experienced? Considering what you have just learned [referring to the position regarding feedback], where do you currently stand? In relation to your learning position, what would you like to improve during your next formative feedback interaction with your supervisor? In relation to humility [openness, tenacity and explanation are addressed in a similar question], what would you like to improve during your next formative feedback interaction with your supervisor? Considering these testimonials and similar situations you may have experienced, what attitudes and behaviors from the H.O.S.T model will you implement during your next interaction with your supervisor?

The interview guide was written in advance by the co-investigators (VL and DBL) and had not been validated in the literature. The questions aimed to explore students’ perceptions of the learning posture and their comfort level with participating in the feedback process after completion of the ASLM. (Table 2) The semi-structured interviews were conducted in French by a co-researcher (VL) and were approximately 15 min long. They were held virtually by audio call on the Microsoft Teams platform, at participants’ convenience.

**Table 2.** List of Semi-Structured Interview Questions.

1. What is your understanding of the learning posture? a. Has this understanding changed this year? If so, what do you attribute that change to? 2. How comfortable are you receiving feedback now? a. Is there anything in particular that brought about this change? 3. Did you notice a difference in how you receive feedback in simulated clinicals (or during clinical rotation) after completing the module? a. Did certain aspects of the module bring about that change? 4. Does the concept of inner speech mean anything? 5. Are you familiar with the concept of investigating a stranger or facing an enemy? 6. The self-learning module was based on the H.O.S.T. model. What do you think of that model?

### Units of Study

The study was conducted among six undergraduate students in the Francophone stream of the University of Ottawa Faculty of Medicine MD program who participate in educational activities (simulated clinicals or clinical rotations) where they receive feedback. Of the six participants in the study, most (*n* = 5) were first-year medical students, with fewer prior opportunities to receive feedback. Only one participant was in their fourth year of medical school and had experienced a greater number of feedback interactions throughout their training.

### Data Processing and Analysis

Data from the semi-structured interviews were analyzed using a thematic analysis, based on the guidelines from Braun and Clarke.^35^ The recorded semi-structured interviews were transcribed verbatim using Microsoft Teams’ automatic transcription feature. The verbatim transcripts were then reviewed by a co-investigator (VL) to ensure accuracy. All statements from the verbatim transcripts relating to participants’ experiences, attitudes and emotions related to feedback were manually and independently coded by the co-investigators, ensuring data triangulation.

### Techniques to Enhance Trustworthiness

To ensure trustworthiness of the analysis, we followed a systematic and collaborative approach. The primary coder first read each transcript multiple times to gain a deep understanding of participants’ perspectives and drafted an initial table of codes grouped into themes based on an interpretative approach. In parallel, the three other members of the research team independently and manually coded the transcripts using the same approach. The team then met to compare interpretations, discuss discrepancies, and refine the coding. A shared table was created to organize individual codes into the categories previously discussed, allowing to identify emerging themes and relevant subthemes with associated quotations. Themes and subthemes were further reviewed against the research objectives, and through successive discussions until consensus was reached.

## Results

Thematic analysis of data from six semi-structured interviews helped identify two main themes, namely reflexivity and engagement, as well as six sub-themes, all related to students’ perceptions of the module and reflections on the feedback process and adopting the learning posture.

### Theme 1: Reflexivity

Based on reflection and self-awareness, reflexivity allows students to put their knowledge into perspective to improve their behaviour and adopt a growth mindset.

#### Sub-Theme 1: Increased Self-Awareness

Following the module, several students described increased awareness of their experiences with feedback. This enabled them to participate more effectively in feedback processes and receive feedback more readily.

“I would say that I view my feedback differently, [the module] reminded me a bit about what the purpose of feedback is […] It's so I can improve.” (MS110)

“If I think about how I received feedback in recent months, I definitely see an improvement. […] I’m more confident asking questions, not just to discard feedback, but to profit from it.” (MS108)

“[…] by being more aware of how we, as students, can try to adapt and take criticism better, I think that just the fact of knowing that, it will already help me take feedback better in my clinicals.” (MS107)

“I think […] I’m really getting better at receiving that feedback without really feeling like it's an attack on me. [It's] much better compared to the beginning of the year.” (MS100)

“[In] my most recent simulated clinicals, I think I was more open to feedback […] The module helped me reflect on how I used to take feedback.” (MS104)

This reflexivity exercise was facilitated by a specific aspect of the module, namely, the opportunity to identify with five fictional students whose attitudes and behaviours place them along the learning-evaluation posture continuum:

“You [could] identify with one of the characters. When you can identify with a character, that makes learning easier […] and you’re able to reflect.” (MS110)

“There were types of residents or medical students associated with a learning style. I think that that really helped to see yourself.” (MS100)

“We were asked, ‘Who do you identify with?’” I think it helped me see that there are lots of people who take feedback differently, that it's normal for it to not always be easy and that, you know, everyone handles it differently […]” (MS104)

#### Sub-Theme 2: Identifying Emotions

The reflection mechanism also helped students recognize their emotional reactions to various aspects of feedback. Some indicated the presence of negative emotions regarding messages received in terms of both their form and their content, but also the positive effect of adopting the learning mindset by accepting mistakes as a source of learning.

“I really think I’m getting better at being able to receive that feedback without feeling too much like it's a personal attack.” (MS100)

“[It's important] to remain humble, to not feel attacked by the person providing the feedback, to accept it, because […] feedback can help us improve.” (MS104)

“[…] And to not let yourself get discouraged […] by the somewhat negative criticism you receive.” (MS107)

“There are preceptors with whom […] you feel you click better, […] you’re able [to apply] what you’ve learned without being stressed about disappointing the preceptor.” (MS107)

“[…] When you’re sure that you’ve done something well, and the preceptor either doesn’t see it or says it was done poorly […] You get a bit closed off to criticism […] and you’re conflicted about the rest of the comments or you’re more closed off or more defensive.” (MS107)

One student explained:

“The reason I wanted to use this module is that [sometimes] when the comments are not necessarily packaged how we like to receive them, it might be more difficult to take them. […] It's not how it's said, it's really looking at the content of what your preceptor is trying to give you.” (MS100)

#### Sub-Theme 3: Inner Speech

The mechanisms of reflexivity and introspection often refer to inner speech as influencers of emotions and behaviour.^27^ The presence of that inner conversation and its power to regulate one's attitudes and behaviours during a feedback situation was presented in the module and was raised by some students.

“But now, the most recent simulated clinicals I had, I think I was more open to feedback. And I tell myself [that] no physician is perfect; it's normal to make mistakes sometimes, that's how you learn.” (MS104)

“I think that it's really much better compared to the beginning of the year […] I do see myself going back to the module and telling myself ‘Okay, try to see […] what they [your preceptor or the resident] are trying to say’ […]” (MS100)

“[…] without feeling as though we’re failing the challenge or the kind of competition we create in our heads as students.” (MS107)

Although the power of inner speech was raised, none of the participants alluded to using the proposed key phrases to facilitate inner conversation as reminders or to adopt each of the components of the H.O.S.T. model (Lifelong improvement, Why not, Staying engaged and Because).

### Theme 2: Actively Engaging in the Feedback Process

The ASLM presented feedback as a reciprocal exchange between the student and the preceptor, fostering a benevolent therapeutic alliance.

#### Sub-Theme 1: Validating Good Practice

Some students reported being comfortable with participating in the student-supervisor interaction, even before completing the ASLM. The module influenced how students perceived and approached feedback, as described in their reflections.

“I always try to take as much as possible from it, ask questions, or make sure that I understand why the other person is telling me this or that […] I already had a good idea of the importance of feedback, so I think that the SLM kind of confirmed its importance. I was able to see where I was, and what I can do to benefit from the feedback even more.” (MS108)

For another student, their comfort with feedback comes from the experience of seeking it to receive it, in another setting. He explained: “I worked before getting into medicine, so I think that in the work environment, it's something you need to seek out [feedback] yourself to be able to improve.” (MS403)

For those students, completing the ASLM validated their good practices, while reminding them how to maximize the benefits of feedback.

#### Sub-Theme 2: Adopting Proactivity

Students who were less familiar with the learning posture were able to validate the importance of participation and initiating conversation based on their needs. They stated their intention to be more proactive in seeking feedback.

“I’m thinking about being more proactive in seeking [feedback], perhaps [by asking] more questions and [asking for] clarification on the feedback I received.” (MS100)

“When I’m at [the hospital] or when I’m in interactive situations, I become the person who needs to seek [what] I need in my learning.” (MS403)

#### Sub-Theme 3: Influence of the Student-Supervisor Dynamic on Proactivity

Some students considered the student-supervisor relationship in a wider context during their reflection and identified legitimate drawbacks to using the H.O.S.T. model. Given that the student-supervisor dyad is part of a complex interpersonal dynamic, the comfort level between student and supervisor varies based on the individuals and the dyads, but also on the moment, as those students stated.

“I also think that the way the criticism is approached depends a lot on the preceptor you have … [With] some preceptors, I would say there is some tension in the air, or [that] stress rises more easily among the students. […] There are preceptors with whom […] you feel it clicks better, that you sense a level of comfort both on the preceptor's side and on ours …]” (MS107)

“It also depends on the person who is giving the feedback, which will determine how comfortable you might be receiving it.” (MS403)

A student would even like to see the bilaterality dimension of the student-supervisor interaction expanded.

“The humility perspective, I understand it, and I agree, but I think it puts a lot of weight on the student. I think it's half and half; the weight should be shared between the student and the preceptor because there are many things that are in the preceptor's hands.” (MS107)

Overall, students reflected on the feedback process through the ASLM, positioning themselves or evolving along the learning-evaluation posture continuum, and recognizing the importance and legitimacy of proactively engaging in the feedback process, while acknowledging that this collaborative process may vary depending on the supervisor and the moment.

## Discussion

The purpose of this study was to explore how students perceived the ASLM, grounded in the H.O.S.T. conceptual model, in relation to raising awareness of the learning mindset and their experience receiving and engaging in feedback with their supervisor. Taking into consideration the small sample, our data suggest that students perceived the ASLM as a promising educational tool that encourages self-regulation by sparking reflexivity and reflection. It provided an opportunity to reflect on the learning posture, supported by a growth mindset, while also offering strategies that students perceived as helpful for receiving feedback more effectively and better engage in the feedback process with their supervisor.

The feedback process is fundamentally a student-supervisor interaction within a particular context in time and space.^7,13,24,26,36–38^ Comments gathered from learners corroborate that the development of feedback literacy among students contributes to informing students about the feedback process and encourages them to engage in the process after reflecting on the criticism.^12^ Moreover, the initiative to focus on the individual in the interactional feedback process and to encourage the development of a personal growth mindset seems to promote students’ openness to better manage their negative emotions regarding mistakes, their impression of disappointing the supervisor or their reaction to a hostile environment.^21^ The perspective of the intrapersonal component was identified as an element that is missing from existing feedback literacy courses.^15^ The opportunity provided in the module for comparison with a fictional peer created a safe environment and promoted reflexivity among the students. Introducing inner speech helped model the thoughts and emotions related to feedback, thereby facilitating the contemplation, the preparation, and for some, the action stages of the behavior change model.^29^

In the context of the learning posture and associated personal growth mindset, some students are now more aware of the two-way component of the interactional feedback process. They would like a more collaborative relationship with their supervisor and propose, as suggested by Richardson et al, that the supervisor adopt the same mindset.^21^ The literature illustrates the importance of longitudinal, honest relationships without hierarchy,^24,37^ and that the quality of the educational alliance is crucial.^7,8^ It was demonstrated that supervisors who model vulnerability by sharing their own challenges, mistakes and failures help learners become more receptive to criticism.^21,24^ Ajjawai et al similarly report that professors who agreed to show their own vulnerability allowed residents to admit their own weaknesses and ask for help.^37^ So, how can we achieve this? Current evidence supports the implementation of institutional programs dedicated to feedback training.^36^ Updating the content and format of courses offered to supervisors to include feedback training sessions for both students and supervisors, as suggested by Nobles et al, may be particularly beneficial.^11^ Such an approach would equip both groups with the attitudes and behaviors associated with a learning posture, and reinforce a culture of continuous improvement.^21^ Although some may question how this kind of practice aligns with supervisors’ evaluative responsibilities, in a context of skills development, Richardson et al suggest that the emphasis would shift from success to progress.^21^ Achieving such a transformation in supervision practices will ultimately require the engagement of students, who are themselves trained to adopt a learning mindset.

### Limitations

We acknowledge several limitations to this study. First, data saturation was not achieved, which limits the completeness of the thematic findings, and suggests that additional perspectives may not have been captured. Additionally, the sampling method and the small sample size do not provide a faithful representation of the first-to-fourth-year medical student population, limiting generalization of the results. Furthermore, the data collection tool was developed by the co-researchers, without being validated, which reduces the reliability of the results and could introduce measurement bias. Finally, the under-representation of clerks participating in clinical rotations with actual patients and receiving feedback from their supervisor regularly limits our ability to explore their experiences and perceptions of the ASLM in the context of higher levels of medical education.

### Future Research

For practical reasons within the scope of this pilot study, the ASLM was delivered a single time to participants in order to explore their perceptions upon completion. In the second phase of this research, the ASLM will be offered across all years of medical education to explore how students perceive and make sense of the learning posture and their comfort engaging with supervisors during the feedback process, taking into account their varying levels of prior feedback experience.

Future research could explore how these same students perceive the ASLM once they enter clerkship, allowing assessment of their progression along the learning-evaluation posture continuum and their engagement in the feedback process. In parallel, this would allow us to explore how students perceive a reinforcement session offered after the initial training, and how this second opportunity supports further reflection on the learning posture and the strategies introduced in the ASLM for receiving feedback effectively.

Consistent with Nobles and al.,^11^ there is a need for feedback literacy training that engage both supervisors and students.^11^ It may be valuable to share the ASLM with supervisors and consider opportunities for joint courses, as a way to further explore how the student-supervisor dyad can be supported.

Finally, it would be interesting to focus on a student population with defensive attitudes and behaviours during feedback and explore how they perceive the use of inner speech as a strategy for emotional self-regulation and for engaging with the learning posture.

## Conclusion

In this pilot study involving six participants, the active self-learning module based on the H.O.S.T. conceptual model was experienced by students as an opportunity for reflective learning on their feedback interactions. Through this exercise, students described becoming more aware of the nature of their engagement in the student-supervisor relationship, reflecting on their place along the learning-evaluation posture continuum, and considering the positive impact of cultivating a personal growth mindset. These findings offer an initial exploration of how students make sense of their attitudes and approaches to feedback. It is a first step in the process of changing attitudes and behaviours related to feedback.

## Supplemental Material

sj-docx-1-mde-10.1177_23821205261423394 - Supplemental material for Promoting the Learning Mindset Among Undergraduate Medical Students: A Qualitative Pilot Study on an Active Self-Learning Module Aimed at Openness During the Feedback Process Supplemental material, sj-docx-1-mde-10.1177_23821205261423394 for Promoting the Learning Mindset Among Undergraduate Medical Students: A Qualitative Pilot Study on an Active Self-Learning Module Aimed at Openness During the Feedback Process by Véronique Lapierre, Anne-Charlotte Côté, Isabelle Burnier and Diane Bouchard-Lamothe in Journal of Medical Education and Curricular Development
